# Comparing the Alteration of Nasal Tip Sensibility and Sensory Recovery Time following Open Rhinoplasty with and without Soft Tissue Removal

**DOI:** 10.1155/2012/415781

**Published:** 2012-11-25

**Authors:** Alireza Bakhshaeekia, Sina Ghiasi-hafezi

**Affiliations:** Division of Plastic and Reconstructive Surgery, Department of Surgery, Shiraz University of Medical Sciences, Shiraz, Iran

## Abstract

In this study we evaluated sensory alteration in nasal tip and adjacent upper columella (territory of external nasal nerve) after open rhinoplasty. Two groups were randomly selected, each containing 25 patients with thick nasal skin; sensory testing was done preoperatively in all patients; in group one, subdermal soft tissue in tip and supratip areas was removed but in group two no soft tissue removal was done; we compared sensory pressure threshold values 3 weeks and 6 months postoperatively. Results showed no statistical difference between the two groups in nasal skin sensibility at these times; also this study showed that 6 months after rhinoplasty normal sensation of nasal skin will be achieved.

## 1. Introduction

Rhinoplasty in patients with thick nasal skin is one of the most challenging operations; regardless of its cause, the thick soft tissue coverage represents a distinct limitation in rhinoplasty because the skin will usually not redrape properly over the nasal skeletal framework [1, 2].

The operative management of such a deformity is controversial and ranges from limited procedures with no soft tissue removal [1] to scoring the dermis [3] or even tip defatting [2–5]. 

An anatomical and histological evaluation of the tissue specimens obtained from the tip and supratip regions showed that collagenous fibrous tissue, adipose tissue, and skeletal muscle were the predominant subdermal tissue type present; fibrous tissue, in fact, comprised the majority of the subdermal tissue so this fibromuscular tissue can be safely resected without violating the dermis thereby decreasing the soft tissue bulk of the bulbous nasal tip and not interfering with the vascular supply to the skin envelope [6]. One of the complications that commonly are seen after rhinoplasty is hypesthesia; the terminal branch of the anterior ethmoidal nerve supplies tip sensation and is usually transected during intercartilaginous incision [7].

In one study 3 weeks and one year after open rhinoplasty the sensibility of various parts of the external nasal skin was evaluated and it was shown that altered sensibility following open rhinoplasty occurs in the early postoperative period (3 weeks post operatively) in the area of skin supplied by the external nasal nerve (nasal tip and adjacent upper columella); but sensation became normal after one year; the nerve is probably injured during the subcutaneous dissection as the nerve passes between the nasal bone and the upper lateral cartilage to supply the skin [8].

According to these studies we decided to evaluate sensory alteration in nasal tip and adjacent upper columella (territory of external nasal nerve), so we randomly selected two groups each containing 25 patients with thick nasal skin; sensory testing was done preoperatively in all patients; in group one we removed subdermal soft tissue, but in group two we did not remove any soft tissue and we compared the sensory recovery in 3 weeks and 6 months postoperatively between these groups with a set of sensory testing monofilaments.

## 2. Patients and Methods

This is a prospective study; 50 patients with thick skinned nose were selected and prepared for open rhinoplasty then randomly divided in two groups (group 1 and group 2), each group containing 25 patients. None on the patients had previous facial surgery or trauma prior to the open rhinoplasty procedure. Furthermore, none had developmental delay, a medical history of diabetes, a systemic neurologic impairment, or a syndrome that might impair sensibility. 

The main indication for surgery was cosmetic improvement of nasal tip. All patients underwent open rhinoplasty using a midcolumellar incision. In group 1 instead of elevating the skin directly over the osteocartilaginous skeleton, in tip and supra tip area, we undermined it under direct vision in a new plane just under the submusculoaponeurotic system extending over collagenous fibrous tissue and adipose tissue that remain attached to the nasal skeleton mostly over the tip and supra tip area, which is dissected off the nasal cartilages and skeleton and discarded; but in group 2 subdermal soft tissue of nasal tip was not touched. 

**Figure 1:**
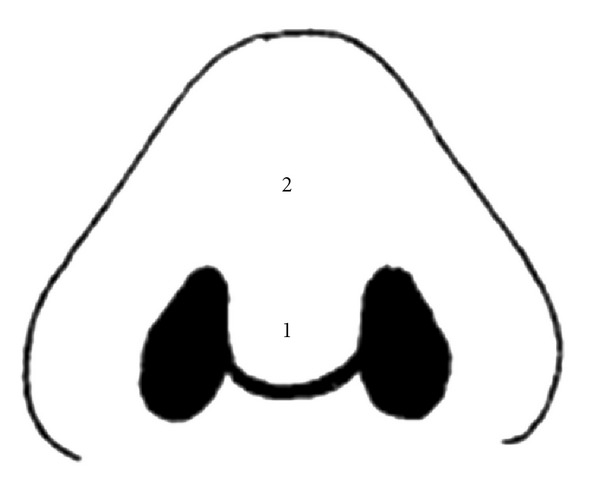
Area determined for objective testing.

All patients underwent tip plasty including resection of the cranial part of the lateral crura of the lower lateral cartilage, interdomal suture, transdomal suture, septal surgery, septal cartilage harvesting, tip graft, and columellar strut graft; most of the patients need spreader graft, all of the patients underwent alar resection. Postoperative recovery was uneventful and no hematoma, infection, or skin necrosis occurred. All patients had subjective questioning and objective testing of nasal sensibility preoperatively, 3 weeks postoperatively, and 6 months postoperatively. Subjectively patients were asked about any alteration of nasal sensibility in tip area; for objective testing two areas were determined as shown in Figure 1. Cutaneous pressure thresholds were tested in each area with a set of Semmes-Weinstein (SW) monofilaments.

**Figure 2:**
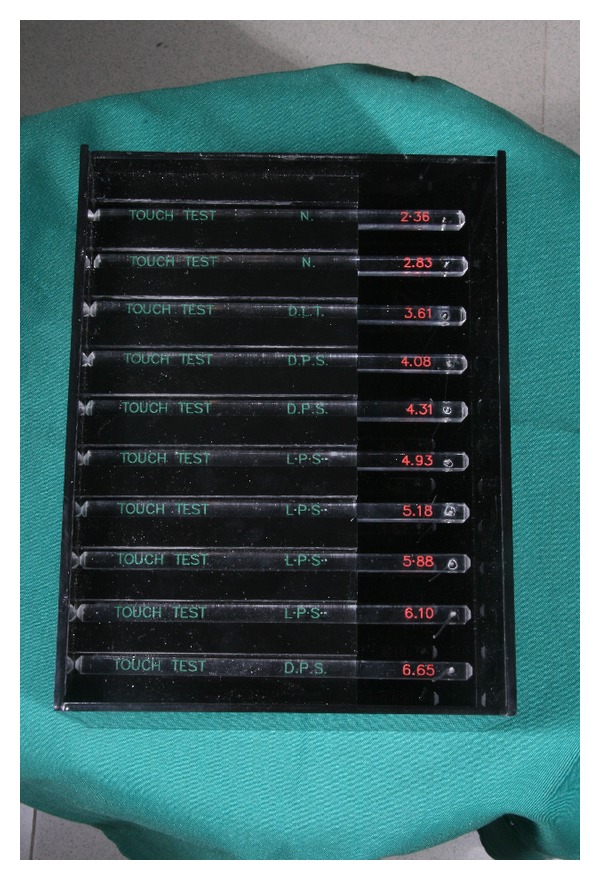
Sensory testing monofilaments.

**Figure 3:**
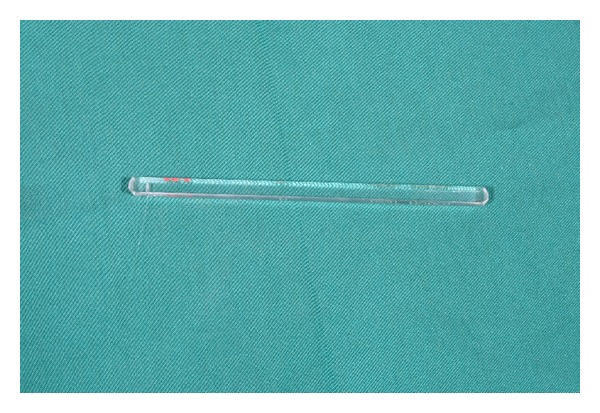
Monofilament no. 2.36.

**Figure 4:**
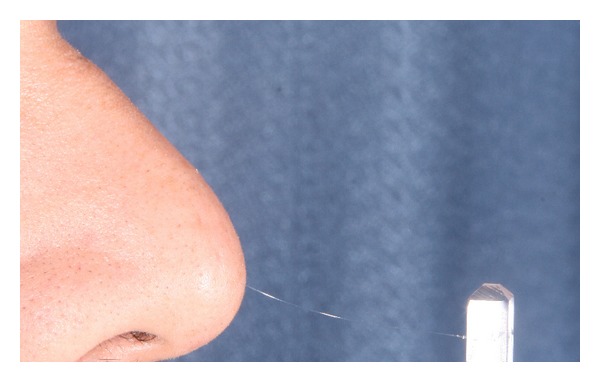
Sensory testing of the nasal tip. The examiner keeps the filament perpendicular to the test site slowly pressing the filament until it bends, holding it on site for about 1.5 seconds.

These SW monofilaments are variable in thickness and labeled with numbers ranging from 2.36 to 6.65 as is shown in Figures 2 and 3, representing the common logarithms of the forces (expressed in 0.1 gram) required bowing the filaments. The examiner keeps the filaments perpendicular to the test site, slowly pressing the filament until it bends, holding it on site for about 1.5 seconds and then lifting it away from the skin (Figure 4). The stress (g/mm^2^) of the lightest filament that evokes perception of pressure is recorded as the pressure threshold, and each area in group one was compared with the same area in group two preoperatively, 3 weeks postoperatively, and 6 months postoperatively. 

## 3. Results

For data analysis we used repeated measures “ANOVA” test and standard deviations were calculated for pressure threshold values at columella (area 1) and tip (area 2) preoperatively, 3 weeks post operatively, and 6 months after surgery; differences between 2 groups were tested using the independent sampled *t*-test.

We had two groups in our study and we compared the role of soft tissue removal in sensory recovery time after open rhinoplasty; in group 1, we performed subdermal soft tissue removal of tip and supratip area and in group 2, we did not remove any soft tissue. Each group contained 25 patients; in group one the patients' ages ranged from 19 to 43 years and mean age was 27.67 years; in group two the patients' ages ranged from 17 to 39 years and mean age was 25.48 years. 

6 patients in group one (24%) and 9 patients in group two (36%) were male.

3 weeks after surgery all patients had subjective alteration in sensibility of the nasal tip and the adjacent upper columellar skin. This alteration was perceived as heaviness in 19 patients in group one and 17 patients in group two; 6 patients in group one and 8 patients in group two stated that the nasal tip and adjacent columella felt different from remaining nasal skin.

Objective testing confirmed the subjective findings and showed higher pressure threshold values 3 weeks after surgery as it is shown in Table 1.

**Figure 5:**
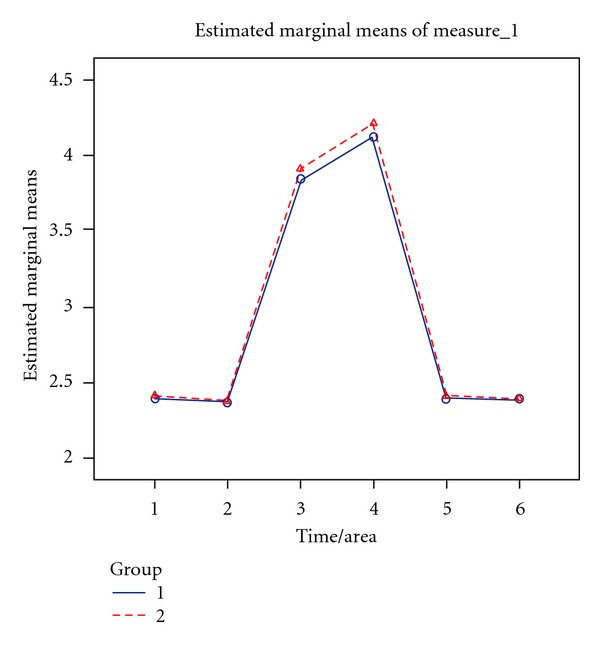
Alteration in pressure threshold measures according to time/area (“time/area” 1 = preoperative/area 1, “time/area” 2 = preoperative/area 2, “time/area” 3 = 3 weeks post op./area 1, “time/area” 4 = 3 weeks post op./area 2, “time/area” 5 = 6 month post op./area 1, “time/area” 6 = 6 month post op./area 2).

Statistical analysis showed no meaningful differences in pressure threshold values between the two groups 3 weeks and 6 months after operation (Figure 5).

## 4. Discussion

In one study [8] they found that altered sensibility following open rhinoplasty occurs in the early postoperative period only in the area of nasal tip (area 3) and adjacent upper columella (area 2); so we select these two area for objective sensory testing in our study (Figure 1) and we decided to perform 2 types of operations and compare these two operations according to nasal skin sensory recovery; open rhinoplasty in group one was accompanied with subdermal soft tissue removal in tip and supratip area and open rhinoplasty in group two was without any subdermal soft tissue removal in nasal tip or supratip area.

Our study confirmed that altered sensibility following open rhinoplasty occurs 3 weeks after surgery in the same area previously reported by Bafaqeeh and Al-Qattan [8]; these areas (nasal tip and adjacent upper columella) are supplied by the external nasal nerve; the nerve is most probably injured during the subcutaneous dissection as the nerve passed between the nasal bone and the upper lateral cartilage to supply the skin; the external nasal nerve injury could also occur during intercartilaginous and cartilaginous splitting incisions of the endonasal rhinoplasty; it is also mentioned in study [8] that they checked nasal sensation one year after operation that showed complete recovery, but we performed nasal sensory testing 6 month after operation and we found that normal sensation and complete recovery is taking place as soon as 6 months postoperatively in nasal skin and there was no difference between two groups, so our study showed that subdermal soft tissue removal did not make any difference in sensory recovery time between two groups 3 weeks and 6 months after surgery.

Although risks of nasal tip soft tissue removal such as skin necrosis or skin irregularity should be noted, complication rate will be decreased if you do not violate the dermis and remain in correct plain; on the other hand Garramone et al. [6] showed that fibrous tissue comprising the majority of subdermal tissue and skeletal muscle was the second most prevalent component of nasal tip subdermal tissue, and it is believed that this fibromuscular tissue can be safely resected without violating the dermis, thereby decreasing the soft-tissue bulk of the nasal tip and not interfering with vascular supply to the skin envelop, and also there is no difference in sensory recovery time of nasal skin “which is a common source of discomfort for rhinoplasty patients” with or without subdermal soft tissue removal in tip and supratip areas.

## Figures and Tables

**Table 1:** Mean and SD of pressure threshold measures according to time/area in each group.

Group statistics					
	Group	*N*	Mean	SD	SEM
Before operation area 1	1	25	2.3976	.13014	.02603
	2	25	2.4164	.15588	.03118
Before operation area 2	1	25	2.3788	.09400	.01880
	2	25	2.3788	.09400	.01880
3 weeks post op. area 1	1	25	3.8444	.27866	.05573
	2	25	3.9160	.34518	.06904
3 weeks post op. area 2	1	25	4.1248	.22309	.04462
	2	25	4.2184	.30461	.06092
6 months post op area 1	1	25	2.3976	.13014	.02603
	2	25	2.4164	.15588	.03118
6 months post op. area 2	1	25	2.3976	.13014	.02603
	2	25	2.3976	.13014	.02603
